# Effects of *Boswellia serrata* resin extract on motor dysfunction and brain oxidative stress in an experimental model of Parkinson’s disease

**Published:** 2019

**Authors:** Parvaneh Doaee, Ziba Rajaei, Mehrdad Roghani, Hojjatallah Alaei, Mohammad Kamalinejad

**Affiliations:** 1 *Department of Physiology, School of Medicine, Isfahan University of Medical Sciences, Isfahan, Iran.*; 2 *Department of Physiology, School of Medicine, Shahed University of Medical Sciences, Tehran, Iran.*; 3 *Department of Pharmacology, School of Pharmacy, Shahid Beheshti University of Medical Sciences, Tehran, Iran.*

**Keywords:** Boswellia serrata, Motor dysfunction, Oxidative stress, Parkinson’s disease

## Abstract

**Objective:**

*Boswellia serrata *oleo-gum resin (frankincense) exerted antioxidant and anti-inflammatory effects against several diseases, such as; asthma, rheumatoid arthritis and irritable bowel syndrome. In the current study, the influences of *B. serrata *resin extract on motor dysfunction and oxidative stress markers were investigated in the intrastriatal 6-hydroxydopamine (6-OHDA) model of Parkinson’s disease (PD).

**Materials and Methods:**

The animals were randomly assigned to sham, lesion (6-OHDA), and three lesion groups treated with ethyl alcoholic extract of *B. serrata *at doses of 125, 250 and 500 mg/kg for 3 weeks. The neurotoxin 6-OHDA (12.5 µg) was microinjected into the left striatum to induce PD in male rats. Motor behavior was assessed by rotational and elevated narrow beam tests. Oxidative stress markers were measured in striatal and midbrain homogenates.

**Results:**

There was a significant increase in contralateral rotations in 6-OHDA group versus sham group (p<0.001), and treatment with *B. serrata *resin extract at doses of 125 and 250 mg/kg significantly decreased the rotations in comparison to 6-OHDA group (p<0.001 and p<0.001, respectively). The 6-OHDA group also showed considerable elevation in the latency to initiate crossing (p<0.001) and the total time (p<0.001) on narrow beam test. Moreover, treatment with *B. serrata* extract at doses of 125, 250 and 500 mg/kg caused a significant reduction in the latency and total time (p<0.001, p<0.001, and p<0.01, respectively). Biochemical analysis showed no significant difference in oxidative stress markers levels among the groups.

**Conclusion:**

Our findings suggest that *B. serrata* resin extract acts as an anti-inflammatory and antioxidant agent that protects nigrostriatal dopaminergic neurons and improve motor impairments in PD.

## Introduction

Parkinson’s disease (PD) is the second most common neurodegenerative disorder with progressive loss of dopaminergic neurons and the presence of Lewy bodies in the substantia nigra pars compacta (SNC) (Rocha et al., 2015 ▶). Bradykinesia, stiffness, resting tremor and postural instability are the most typical symptoms of PD (Wang et al., 2015 ▶). The typical age is 50-60 years, but young adults and children can also be affected by this disease (Zafar et al., 2003 ▶). The prevalence of PD is 315 in 100,000 people, but this rate increases with age (Pringsheim et al., 2014 ▶). Incidence of PD for males is around two times more than that of females (Pringsheim et al., 2014 ▶). The exact etiology of PD still remains to be identified, but oxidative stress (Blesa et al., 2015 ▶), neuroinflammation (Rocha et al., 2015 ▶), mitochondrial dysfunction (Ryan et al., 2015 ▶) and apoptosis (Ziv et al., 1998 ▶) all contribute to neuronal loss in PD. A progressive production of reactive oxygen species (ROS) which exceeds cellular antioxidant activity, causes oxidative stress (Brieger et al., 2012 ▶). High quantity of ROS causes toxic reactions leading to necrotic and apoptotic death (Phaniendra et al., 2015 ▶). The presence of high levels of unsaturated fatty acids, low levels of antioxidants and high amounts of iron in some areas of the brain, such as; substantia nigra and globus pallidus make these areas susceptible to oxidative stress damage (Sian‐Hülsmann et al., 2011 ▶). Evidence suggests that in the PD brain, ROS and reactive nitrogen species contribute to protein oxidation and nitration, DNA fragmentation and lipid peroxidation (Tsang and Chung, 2009 ▶). 

Chronic neuroinflammation is also an important cause of neuronal loss in PD, which is primarily related to activation of microglia and, to a lesser extent, astrocytes and oligodendrocytes (Blesa et al., 2015 ▶). Activated microglia was found in the SNC, putamen, hippocampus and olfactory bulb of PD patients (Rocha et al., 2015 ▶; Doorn et al., 2014 a,b ▶). Cerebrospinal fluid and postmortem brains of PD patients also displayed elevated levels of the proinflammatory cytokines like tumor necrosis factor- (TNF) α, interleukin- (IL-) 1β and IL-6 (McCoy et al., 2006 ▶; Ferrari et al., 2006 ▶; Rocha et al., 2015 ▶). Chronic release of pro-inflammatory cytokines by activated microglia leads to the exacerbation of dopaminergic neuron degeneration in the SNC. 

*Boswellia serrata* is a tree of Burseraceae family, which grows in dry mountainous regions of India, Northern Africa and the Middle East (Siddighi, 2011 ▶). The oleo-gum resin of *B. serrata* has been used as an effective remedy for headache (Dalla Libera et al., 2014 ▶), colitis (Gupta et al., 2001 ▶), arthritis (Sander et al., 1998 ▶) and inflammation (Darshan and Doreswamy, 2004 ▶). Recent scientific reports indicated the ability of *B. serrata* as a potent antioxidant (Beghelli et al., 2017 ▶; Kokkiripati et al., 2011 ▶) and anti-inflammatory (Siddighi, 2011 ▶; Ammon, 2006 ▶) agent, and a modulator of immune system (Ammon, 2010 ▶). It is also effective in reducing brain edema accompanying glioma (Winking et al., 2000 ▶). The pharmacological effects of *B. serrata *resin extract are mainly attributed to boswellic acids, especially 11-keto-β-boswellic acid (KBA) and acetyl-11-keto-β-boswellic acid (AKBA) (Abdel-Tawab et al., 2011 ▶).

The aim of the current study was to investigate the beneficial effects of *B. serrata *resin extract at doses of 125, 250 and 500 mg/kg on motor dysfunction and oxidative stress markers in a 6-OHDA model of PD.

## Materials and Methods

**Preparation of** ***B. serrata*** **extract**

*B. serrata *extract was prepared in Faculty of Pharmacy, Shahid Beheshti University of Medical Sciences (Tehran, Iran). The golden brown small pieces of oleo-gum resin of *B. serrata *(named, olibanum with an aromatic scent) were purchased from the herbal Pharmacy, and was graciously identified by Tehran University herbarium (Tehran, Iran). Oleo-gum resins were rinsed, dried and powdered. Then, 2000 ml ethanol (96%) was added to 500 g of oleo-gum resin in an Erlenmeyer. The mixture was covered with foil and left for maceration for 48 hr at room temperature. Then, it was filtered and evaporated by a rotary evaporator. The ethyl alcoholic extract of *B. serrata *was kept in a covered glass bottle, in darkness, at room temperature. 

**Animals**

Wistar rats (adult male, 200-250g), procured from Pasteur’s Institute (Tehran, Iran) were used. Animals were retained in a room with adjusted temperature and 12 hr- 12 hr dark-light cycles, and they had free access to food and water. The Ethics Committee for Animal Experiments at Isfahan University of Medical Sciences approved the study (Approval No. 395809) and all experiments were conducted in accordance with the National Institute of Health Guide for the Care and Use of Laboratory Animals (NIH Publication, 8th edition, 2011).

The animals were randomly assigned to five groups as follows: sham group (n=7), lesion group (6-OHDA, n=7), and three lesion groups treated with *B. serrata *extract at doses of 125, 250 and 500 mg/kg (6-OHDA+BS 125, 250, or 500, n=6-7). The extract was injected intraperitoneally from 14 days before to 7 days after the surgery. The neurotoxin 6-OHDA was microinjected via a 10-μl Hamilton syringe into the left striatum of the animals that were anaesthetized by a mixture of ketamine/xylazine (80/5mg/kg, ip) and supported by a stereotaxic apparatus (Stoelting, USA). 6-OHDA was injected at the coordinates: AP+0.2mm, L−3mm, V4.5mm (Paxinos and Watson, 2005). The lesion groups were microinjected slowly (1 µl/min) with a single dose of 12.5 µg 6-OHDA (Sigma Aldrich, USA) in 5 µl of 0.2 % ascorbic acid (w/v) and normal saline. The needle remained in place for 5 min, and then was slowly withdrawn at a rate of 1 mm/min. Sham group received a single dose of ascorbate-saline solution into striatum. 

**Movement Analysis**

The movement analysis was performed one week after the surgery. Trials were done between 10:00 a.m. and 17:00 p.m. The experiment was carried out under normal conditions. 

**Rotational behavior**

The rats were examined for rotational movement using a previously described method (Fujita et al., 1996 ▶). After spending 10 min for animals’ adaptation, and 1 min after injection of 2 mg/kg apomorphine hydrochloride, the rotational behavior was evaluated in a barrel-shaped box with 35 cm in height and 33 cm in diameter. Total rotations were counted at 10 min intervals for 60 min in a dimly-lit and quiet room. The number of ipsilateral rotations was recorded as negative scores and the number of contralateral rotations as positive scores. Net number of rotations was defined as the difference in the scores of rotations in the two directions.

**Elevated narrow beam test**

The narrow beam used in the present study was a wooden beam (length: 105 cm, width: 4cm, and thickness: 3 cm). The beam was elevated 80 cm from the ground and the initial 20 cm of the beam was defined as starting zone. A platform was placed at the opposite end of the beam allowing animals to exit. A mattress was located beneath the beam to protect animals from injury due to falling from the beam. During the test, animals were placed within the starting zone and the time spent to cross the start line, was recorded. This time period represented the latency to begin the task. Also, the time for crossing the beam was recorded. The maximum time period (i.e. cut-off) allowed for the task was 2 min. Duration of time period required for passing the starting zone must not exceed 1min; otherwise, the trial was cancelled and maximum time was recorded for that trial. A fall was also recorded as a maximum time. A testing session which consisted of five trials on the beam, recording five latencies to begin the test, and five total times on the beam for each animal, was held (Allbutt and Henderson, 2007 ▶).

**Biochemical assay**

One week after the surgery, animals were sacrificed and brains were quickly removed. For biochemical assays, striatum and midbrain were dissected out, homogenized in cold saline 0.9% and centrifuged at 4°C. The creamy supernatant was obtained and kept at -80°C until being assayed. 

**Midbrain and striatal malondialdehyde (MDA) assay**

The MDA levels of the supernatant were measured as previously explained (Roghani et al., 2010 ▶). Briefly, trichloroacetic acid and thiobarbituric acid were added to the supernatant, and then, the mixture was heated in a boiling water bath for 90 min. Afterwards, samples were cooled and centrifuged at 1000 rpm for 10 min. The absorbance of the supernatant was read at 532 nm. Standard curves were prepared using tetraethoxypropane.

**Midbrain and striatal glutathione (GSH) assay**

Measurement of GSH levels was performed by a previously described procedure (Sedlak and Linsay, 1968 ▶). The supernatant was centrifuged with 5% trichloroacetic acid to centrifuge out the proteins. To 0.1 ml of homogenate, 2 ml of phosphate buffer (pH 8.4), 0.5 ml of DTNB (5,5' dithio-bis-2-nitro benzoic acid) and 0.4 ml of distilled water were added and the absorbance was read at 412nm. 

**Midbrain and striatal catalase assay**

Claiborne’s method was used to determine catalase activity (Claiborne, 1985 ▶). Briefly, hydrogen peroxide (H_2_O_2_) was added to a mixture, which contained supernatant and potassium phosphate buffer (50 mM, pH 7.0). Then, the rate of H_2_O_2_ decomposition was assessed by recording the absorbance variations at 240 nm for 2 min. One unit of catalase activity was defined as 1 μM of H_2_O_2_ which was decomposed in 1 min.

**Statistical analysis**

Data are presented as mean±SEM. The one-way analysis of variance (ANOVA) followed by Tukey’s *post hoc* test was used for statistical analysis. A statistical p-value <0.05 was considered significant. 

## Results

**Rotational behavior in** ***B. serrata*** **-treated groups**

**Figure 1:**
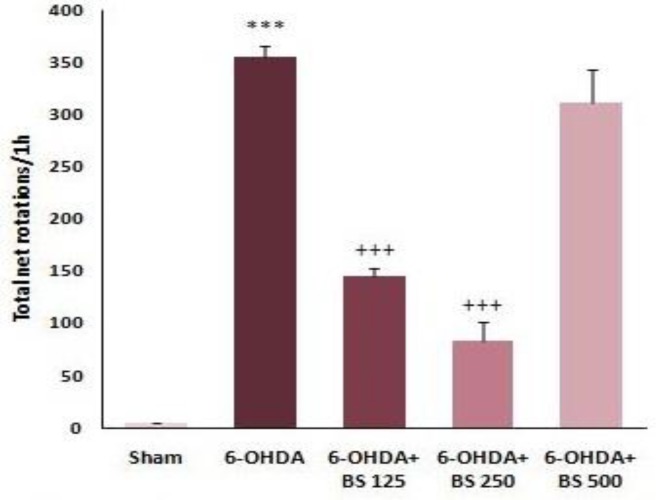
The net number of rotations following apomorphine injection one week after intrastriatal microinjection of 6-OHDA to the sham, 6-OHDA and *Boswellia serrata *(BS)*-*treated groups (125, 250 and 500 mg/kg for 3 weeks). Data are presented as mean±SEM. n=6-7 in each group. ***p<0.001 shows significant difference vs sham group and +++p<0.001 shows significant difference vs 6-OHDA group

The net number of rotations was measured 1 min after injection of 2 mg/kg apomorphine hydrochloride. There was a significant increase in contralateral rotations in 6-OHDA group versus sham group (p<0.001, Figure 1). In addition, the net number of rotations in 6-OHDA+BS 125 and 250 groups significantly decreased in comparison to 6-OHDA group (p<0.001 and p<0.001, respectively; Figure 1). However, there was no meaningful difference between 6-OHDA+BS 500 and 6-OHDA groups (Figure 1).

**Narrow beam test in** ***B. serrata*** **-treated groups**

**Figure 2:**
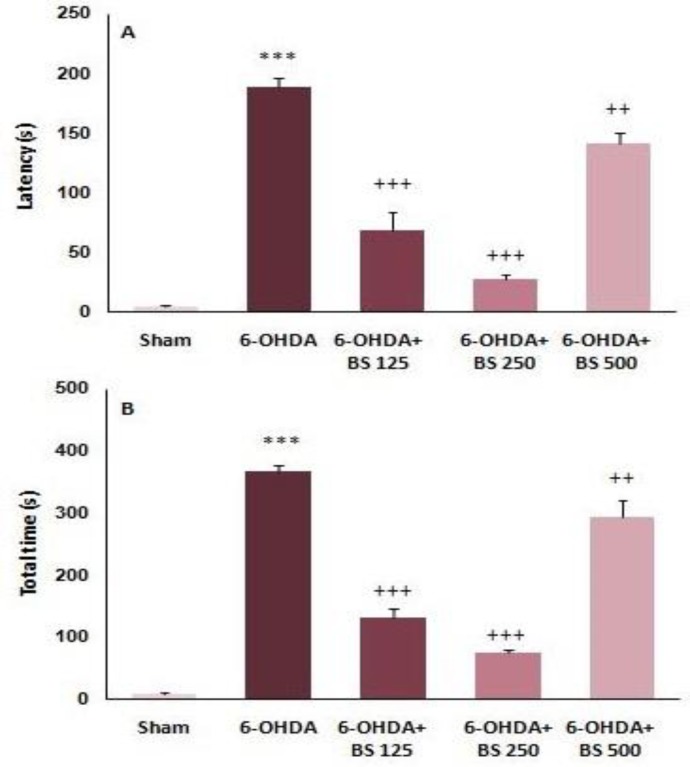
The latency to initiate crossing (A) and the total time to cross the beam (B) in narrow beam test one week after intrastriatal injection of 6-OHDA to the sham, 6-OHDA and *Boswellia serrata *(BS)-treated groups (125, 250 and 500 mg/kg for 3 weeks). Data are presented as mean±SEM. n=6-7 in each group. ***p<0.001 shows significant difference vs sham group; ++P<0.01 and +++ p<0.001 shows significant difference vs 6-OHDA group

One week after the surgery, narrow beam test was performed. Latencies to start the crossing and total times in narrow beam test were recorded. The 6-OHDA group showed considerable elevations in the latency (p<0.001, Figure 2A) and total time (p<0.001, Figure 2B) as compared to sham group. Moreover, 6-OHDA+BS 125, 250, and 500 groups indicated remarkable reductions in latency (p<0.001, p<0.001, and p<0.01, respectively; Figure 2A) and total time (p<0.001, p<0.001, and p<0.01, respectively; Figure 2B) as compared to 6-OHDA group.

**MDA levels in** ***B. serrata*** **-treated groups**

**Figure 3:**
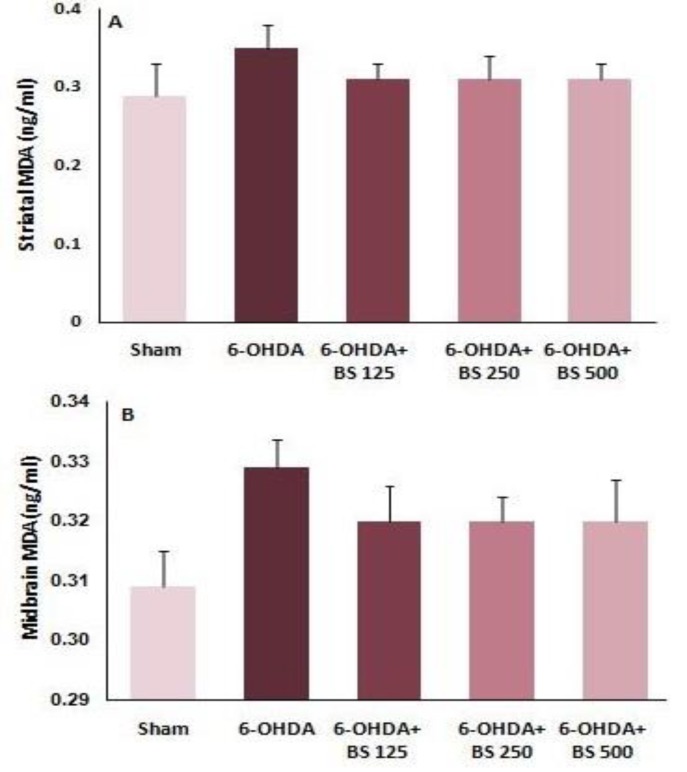
Malondialdehyde levels in the striatum and midbrain one week after intrastriatal injection of 6-OHDA to the sham, 6-OHDA and *Boswellia serrata *(BS)-treated groups (125, 250 and 500 mg/kg for 3 weeks). Data are presented as mean±SEM. n=6-7 in each group

One week after the surgery, striatum and midbrain were dissected out and the supernatant was produced and kept at -80°C for biochemical assessment. Striatal and midbrain MDA levels were measured. The results showed that there was no significant difference in striatal and midbrain MDA levels among groups one week after the surgery (Figures 3A and B).

**GSH levels in** ***B. serrata*** **-treated groups**

**Figure 4:**
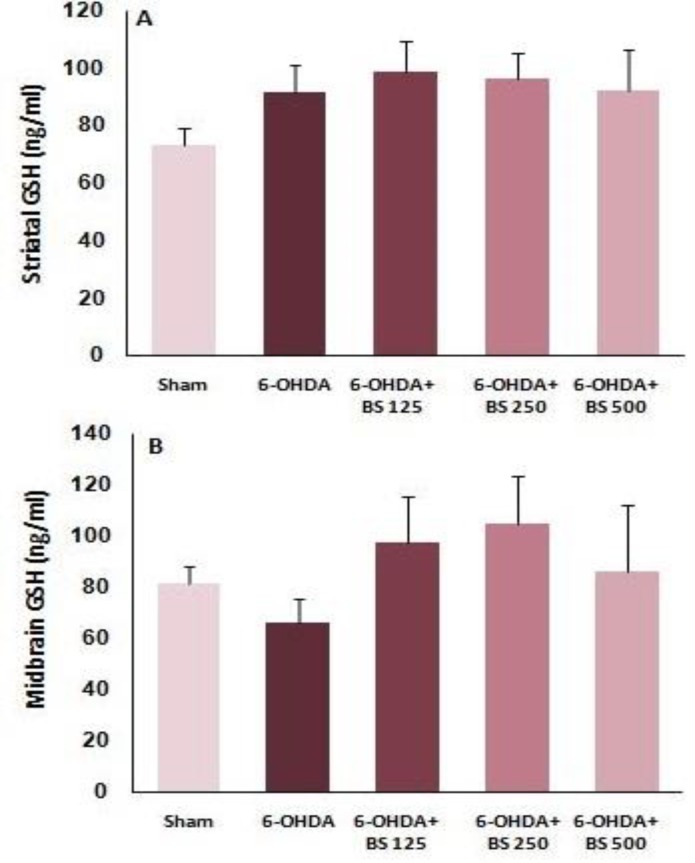
Glutathione levels in the striatum and midbrain one week after intrastriatal injection of 6-OHDA to the sham, 6-OHDA and *Boswellia serrata *(BS)-treated groups (125, 250 and 500 mg/kg for 3 weeks). Data are presented as mean±SEM. n=6-7 in each group

Striatal and midbrain GSH levels were measured. Data analysis showed that there was no significant difference in striatal and midbrain GSH levels among the groups one week after the surgery (Figures 4A and B).

**Catalase activity in** ***B. serrata-*** **treated groups**

**Figure 5:**
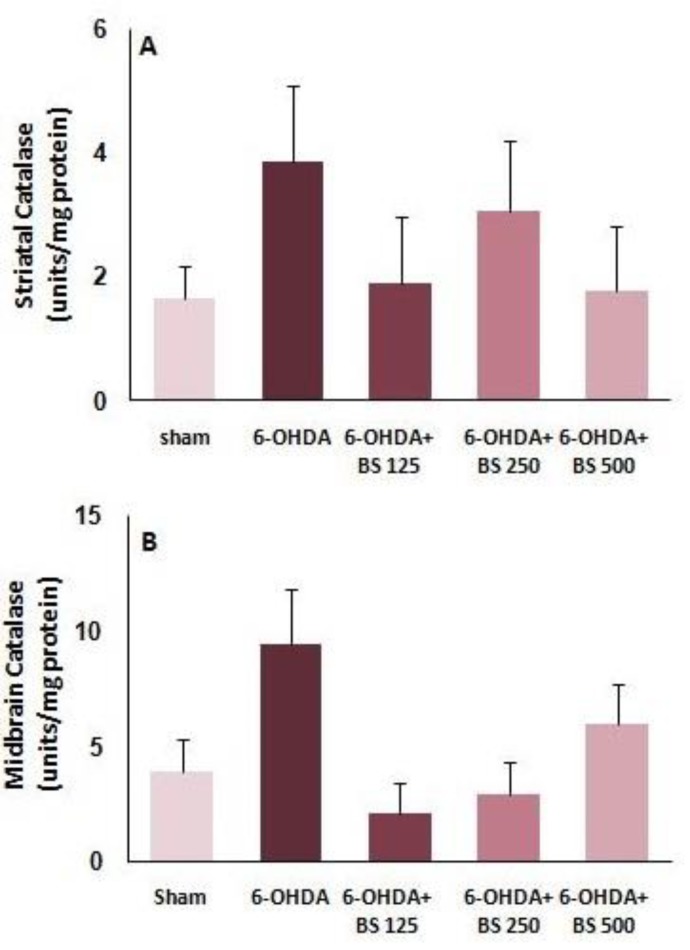
Catalase activity in the striatum and midbrain one week after intrastriatal injection of 6-OHDA to the sham, 6-OHDA and *Boswellia serrata *(BS)-treated groups (125, 250 and 500 mg/kg for 3 weeks). Data are presented as mean±SEM. n=6-7 in each group

Catalase activity was determined via Claiborne’s method. Statistical analysis showed no significant difference in striatal and midbrain catalase activity among experimental groups one week after surgery (Figures 5A and B).

## Discussion

Our findings indicated that microinjection of 6-OHDA into striatum caused a significant increase in contralateral rotations, as well as a considerable elevation in the latency to initiate crossing and the total time in narrow beam test. Moreover, pretreatment with *B. serrata *resin extract at applied doses, significantly decreased the rotations and caused a significant reduction in the latency and total time. However, there was no significant difference in oxidative stress markers levels among the experimental groups.

The neurotoxin 6-OHDA is commonly used to induce PD, because dopamine transporter (DAT) can easily transport 6-OHDA into dopaminergic neurons and cause toxicity and neurodegeneration (Wang et al., 2013 ▶). The intrastriatal injections of 6-OHDA induce a direct toxic damage to the dopaminergic axons in the striatum, which is followed by a gradual loss of dopaminergic neurons in the substantia nigra (Kirik et al., 1998 ▶). Unilateral injection of 6-OHDA into striatum leads to reduction of striatal dopamine levels and upregulation of dopamine receptors, which produce motor asymmetry that can be evaluated by dopamine agonists, such as apomorphine (Schwarting and Huston, 1997 ▶). Rotational asymmetry is used as a characteristic behavioral sign of striatal dopamine loss in unilateral animal models of PD (Henderson et al., 2003 ▶). In the present study, significant contralateral rotations were observed in 6-OHDA-lesioned rats, which is consistent with previous studies (Smith and Cass, 2007 ▶; Baluchnejadmojarad et al., 2009 ▶; Hosseini et al., 2016 ▶; Haddadi et al., 2018 ▶). Our findings also showed that pretreatment with *B. serrata* resin extract administered at doses of 125 and 250 mg/kg for 3 weeks, significantly reduced the contralateral rotations. This could be mainly attributed to the ability of *B. serrata* extract to preserve dopaminergic neurons and maintain the striatal dopamine levels in the optimal levels. The neuroprotective effect of *B. serrata* resin extract against MPP+- induced neuronal death in dopaminergic cell line, SK-N-SH, was reported (Kazmi et al., 2011 ▶). In addition, Ding and colleagues (2014) ▶ showed that acetyl 11-keto-ß-boswellic acid (AKBA) - an active triterpenoid compound from *B. serrata *resin*- *protects neurons against ischemic injury (Ding et al., 2014 ▶). 

The results also demonstrated that the latency and total time in narrow beam test, were increased in 6-OHDA-lesioned rats in comparison to sham rats. In other words, the lesioned rats showed delay in initiating the task and had a lower speed in crossing the beam, which indicate the sign of bradykinesia and/or akinesia (Allbutt and Henderson, 2007 ▶). These signs in hemiparkinsonian rats could also be due to dopamine depletion in the striatum. According to our results, treatment with *B. serrata* extract at doses of 125, 250 and 500 mg/kg was able to correct motor abnormalities in beam test, confirming the neuroprotective effect of *B. serrata* extract in a 6-OHDA-induced model of PD. 

Previous studies reported that 6-OHDA causes a loss in dopaminergic neurons by formation of various oxidants and free radicals, protein carbonyls, lipid peroxidation, and depletion of reduced glutathione (Smith and Cass, 2007 ▶; Khuwaja et al., 2011 ▶; Ahmad et al., 2005b ▶). *B. serrata* extract was shown to modulate oxidative stress status (lipid peroxidation, GSH, catalase, superoxide dismutase, and nitrite oxide levels) in arthritis (Umar et al., 2014 ▶). Moreover, the antioxidant and free radical scavenging activity of *B. serrata *extract was reported by several *in vitro* studies (Singh et al., 2012 ▶; Beghelli et al., 2017 ▶). Thus, the neuroprotective effect of *B. serrata *extract could be partly due to its antioxidant activity. 

However, our results showed no significant difference in oxidative stress markers levels among experimental groups, one week after the surgery. For explanation of this observation, we refer to Smith and colleagues’ study (2007) ▶ which reported increases in the amount of two oxidative stress indicators, 4-hydroxynonenal (HNE, a product of lipid peroxidation) and protein carbonyls, in the striatum just on the first day of 6-OHDA injection, while it reduced to its initial value within 7 days, but dopamine did not raise to normal and remained at lower levels until day 7 (Smith and Cass, 2007 ▶). In our research, the brains were removed one week after 6-OHDA lesioning. It is possible that non-significant variations among the groups in terms of biochemical parameters (MDA, GSH, and catalase activity) can be due to the above-noted findings that these markers return to basal levels after 7 days, the same as other oxidative stress markers, HNE and protein carbonyls, even though dopamine reduction exists at this time. Taken together, it could be concluded that the improvement of motor behavior could be due to antioxidant activity of *B. serrata* against early 6-OHDA lesioning. 

Moreover, neuroinflammation also plays an important role in degeneration of nigrostriatal neurons in PD (Rocha et al., 2015 ▶; Wang et al., 2015 ▶). Post-mortem analyses of PD patients revealed the presence of activated microglia expressing inflammatory cytokines like TNF-𝛼 and IL-6, as well as enzymes associated with inflammation, such as inducible isoform of nitric oxide synthase (iNOS) and cyclooxygenase-2 in the brain tissue of patients (Rocha et al., 2015 ▶). Chronic release of pro-inflammatory cytokines such as TNF-α, IL-1β and IL-6, leads to neuronal loss in the SNC by activating signaling pathways involved in mitochondrial toxicity, caspase-dependent apoptosis and other forms of cell loss (Rocha et al., 2015 ▶; Wang et al., 2015 ▶). It was reported that oral administration of *B. serrata* gum resin extract resulted in significantly reduced levels of inflammatory mediators (TNF-α, IL-1β and IL-6), and increased levels of IL-10 in local tissue in rheumatoid arthritis (Umar et al., 2014 ▶). In addition, *B. serrata* extracts and boswellic acids were shown to inhibit activation of nuclear factor-kappa B and consequently cause a down regulation of TNF-α and interleukins (Ammon, 2010 ▶). Therefore, the neuroprotective activity of *B. serrata* extract in our study, could also be mediated via its anti-inflammatory activity.

In summary, the present study suggests that *B. serrata* resin extract possibly acts as an anti-inflammatory and antioxidant agent, protects nigrostriatal dopaminergic neurons and improves the abnormal behaviors and motor asymmetry in PD.
